# The Therapeutic Potential of Targeting BARF1 in EBV-Associated Malignancies

**DOI:** 10.3390/cancers12071940

**Published:** 2020-07-17

**Authors:** Angela Kwok-Fung Lo, Christopher W. Dawson, Hong Lok Lung, Ka-Leung Wong, Lawrence S. Young

**Affiliations:** 1 Department of Chemistry, Hong Kong Baptist University, Kowloon Tong, Hong Kong SAR, China; kfloaangela@hkbu.edu.hk; 2 Warwick Medical School, University of Warwick, Coventry CV4 7AL, UK; C.Dawson.3@warwick.ac.uk; 3 Department of Biology, Hong Kong Baptist University, Kowloon Tong, Hong Kong SAR, China; hllung2@hkbu.edu.hk

**Keywords:** EBV, EBV-associated cancers, BARF1, immunotherapy, peptide therapy

## Abstract

Epstein-Barr virus (EBV) is closely linked to the development of a number of human cancers. EBV-associated malignancies are characterized by a restricted pattern of viral latent protein expression which is sufficient for the virus to both initiate and sustain cell growth and to protect virus-infected cells from immune attack. Expression of these EBV proteins in malignant cells provides an attractive target for therapeutic intervention. Among the viral proteins expressed in the EBV-associated epithelial malignancies, the protein encoded by the BamHI-A rightward frame 1 (BARF1) is of particular interest. BARF1 is a viral oncoprotein selectively expressed in latently infected epithelial cancers, nasopharyngeal carcinoma (NPC) and EBV-positive gastric cancer (EBV-GC). Here, we review the roles of BARF1 in oncogenesis and immunomodulation. We also discuss potential strategies for targeting the BARF1 protein as a novel therapy for EBV-driven epithelial cancers.

## 1. Introduction

Epstein-Barr Virus (EBV) is a human gamma herpesvirus that infects more than 95% of the population worldwide. EBV persists lifelong in the human host by adopting a latent form of infection with periodic episodes of lytic replication. Upon initial infection of submucosal B-lymphocytes in the naso/oropharynx, EBV establishes a latent infection in memory B cells, with infected cells expressing a limited subset of viral latent proteins that allow the virus to persist while evading host immune attack [1,2].

Primary EBV infection in children is usually asymptomatic; however, EBV may cause infectious mononucleosis (IM) when primary infection occurs in adolescents and young adults. Although rarely pathogenic in the majority of individuals, EBV is commonly associated with posttransplant and AIDS-associated lymphoproliferative disease (PTLD) in immunosuppressed patients. EBV is also linked to the development of several lymphomas and carcinomas in immunocompetent individuals. These include endemic Burkitt’s lymphoma (BL), Hodgkin’s lymphoma (HL), Non-Hodgkin lymphoma (NHL), NK/T cell lymphoma (NKTL) as well as nasopharyngeal carcinoma (NPC), and a subset of gastric carcinomas (GC). In total, EBV-associated malignancies account for approximately 1.8% of all cancer deaths worldwide [1,2,3]. 

Standard treatments for EBV-associated epithelial malignancies include radiotherapy, in combination with various chemotherapeutic drugs. Although the overall survival rates of patients diagnosed with early malignancies are encouraging, there are significant rates of treatment failure in patients with more advanced disease. Also, the long-term posttreatment toxicities are a substantial burden for patients [4,5]. The limitations of current treatment protocols have prompted the development of more effective therapies. Given the strong association of EBV with these malignancies, EBV-encoded proteins expressed within the tumour have been considered as viable therapeutic targets. In this regard, the secreted BamHI-A Rightward Frame 1 (BARF1) protein, which is selectively expressed in EBV-associated epithelial malignancies, is of particular interest. Here, we describe the potential of targeting the BARF1 protein as a novel therapeutic strategy for EBV-associated cancers.

### EBV Latent Genes Expression in Cancer

EBV can adopt various forms of latency (latencies I, II and III), which differ in their repertoire of latent proteins expressed and are largely determined by host cell factors. In vitro, EBV efficiently infects and transforms primary B cells into lymphoblastoid cell lines (LCLs). Both LCLs and PTLD display type III latency, where all latent proteins are expressed. This includes six EBV Nuclear Antigen Proteins (EBNA-1, -2, -3a/3b/c and -5), three Latent Membrane Proteins (LMP1 and LMP2a/b), various noncoding RNAs: EBV-encoded small RNA (EBER1, EBER2) and BamHI-A Rightward Transcript (BART) RNAs, as well as the BamHI-H Rightward Frame 1 (BHRF1) miRNAs and BART miRNAs. HL, NHL, NKTL and NPC exhibit type II latency, where viral gene expression is limited to EBNA1, EBER1/2, LMP1, LMP2a/b, the BART RNAs and miRNAs. EBV-associated GC and endemic BL exhibit type I latency, expressing only EBNA1, LMP2a, EBER1/2, and the BART RNAs and miRNAs. These forms of EBV latency in tumours are very fluid, and there is increasing evidence that proteins associated with the virus’s replicative cycle can also be expressed in virus-associated tumours [1,2,3]. 

## 2. The EBV-Encoded BARF1 Protein

Interestingly, the BamHI-A Rightward Fame-1 (BARF1) protein is exclusively expressed as a latent gene in EBV-positive epithelial tumour cells. BARF1 transcripts are abundantly detected in the tissues of NPC and EBV-associated GC [6,7,8,9]. ΔNp63α, an epithelial-specific transcription factor expressed in undifferentiated NPC cells, has been shown to transactivate the BARF1 promoter to regulate constitutive expression in EBV-positive epithelial malignancies [10]. In B cells, BARF1 expression is restricted to viral lytic replication. BARF1 expression is generally undetectable in latently infected lymphoid malignancies, although some studies have documented expression of BARF1 in Malawian cases of BL and NKTL [9,11,12]. BARF1 is an early lytic protein in B cells and a target of the immediate-early transactivator protein BRLF1. BRLF1 binds directly to multiple R-responsive elements of the BARF1 gene and stimulates its expression during the early phase of the lytic cycle [13,14].

**Figure 1:**
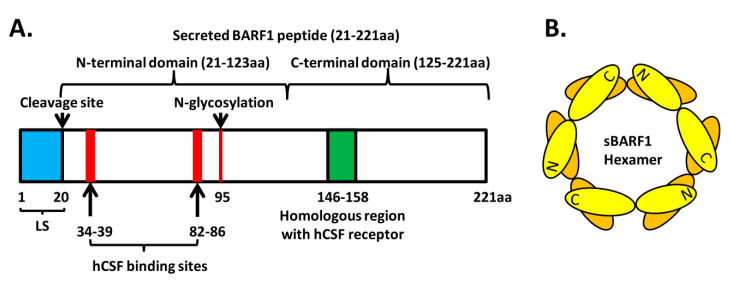
Schematic representation of the BamHI-A rightward frame 1 (BARF1) protein: (**A**) A diagram showing the positions of the leader sequence (LS), cleavage site, N-linked glycosylation site, human colony-stimulating factor (hCSF) binding sites and the homologous region with an hCSF receptor. (**B**) The soluble BARF1 hexameric structure: It is composed of three dimer BARF1 molecules which are interconnected N-terminal domain (head) to C-terminal domain (tail) and are arranged in two layers.

The BARF1 gene encodes a 220 amino acids protein (Figure 1). Immunofluorescence staining and western blotting analysis performed on EBV-infected cell lines induced into EBV lytic cycle or cells transfected with a BARF1 expression vector identified BARF1 as a cytoplasmic and membrane-associated polypeptide [15,16,17,18]. A later crystallographic study has revealed that BARF1 is a soluble hexameric protein molecule [19]. After synthesis in the endoplasmic reticulum (ER), the mature BARF1 protein undergoes posttranslational modification in the Golgi apparatus with the addition of high mannose N-linked glycosylation at the Asn95 residue. The N-linked glycosylation is essential for folding and secretion. After cleaving the 20-amino-acid membrane-associated peptide leader sequence, BARF1 is secreted as a soluble hexameric glycosylated complex. A secreted 29 kDa BARF1 protein molecule consists of two immunoglobulin (Ig)-like domains. The N-terminal domain ranging from residues 21 to 123 is similar to the subfamily of variable domains and the C-terminal domain from residues 125 to 220 is associated with a constant Ig-domain. The secreted BARF1 hexameric protein complex is composed of three dimer BARF1 molecules which are interconnected head to tail and are arranged in two layers [19,20]. 

### 2.1. The Immunomodulatory Properties of BARF1

**Figure 2:**
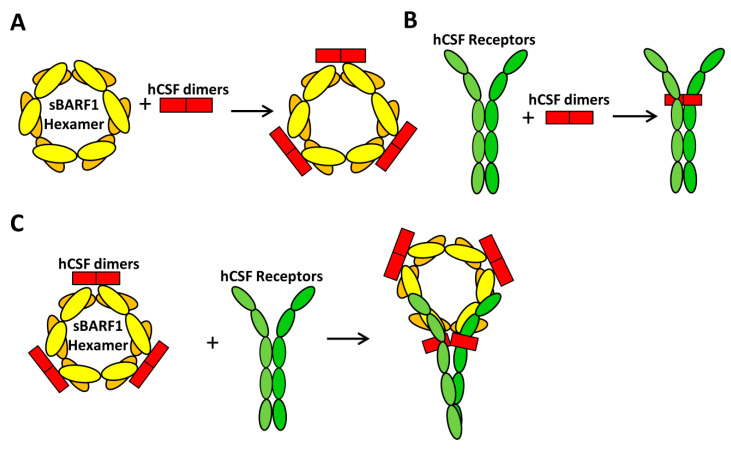
The binding of secreted BARF1 (sBARF1) with hCSF1 reduces the interaction of hCSF1 with its cognate receptor. (**A**) The interaction of one sBARF1 hexamer with three hCSF1 dimers. (**B**) The binding of hCSF dimers with its cognate receptor, CSF1R/CD115. (**C**) The binding of sBARF1 with hCSF1 causes an inactive conformation of hCSF1, hindering the interaction with its cognate receptor.

The secreted BARF1 (sBARF1) protein has been shown to possess immunomodulatory functions. BARF1 was found to be a homolog of the human c-fms protein, the receptor for human colony-stimulating factor 1 (hCSF1), and the homolog domain is located between amino acids 146 and 158 of BARF1 (Figure 1) [21]. hCSF1, also known as macrophage CSF (M-CSF), regulates the survival, proliferation and differentiation of mononuclear cells from monocytes to macrophages; it also stimulates the secretion of alpha interferon (IFN-α) from mononuclear cells. Structural analysis indicates that the hexameric sBARF1 protein interacts with three hCSF1 dimers through residues 34–39 and residues 82–86 located in the protruding N-terminal loops of sBARF1 distant from the cognate receptor-binding site (Figure 1 and Figure 2) [22,23,24]. The binding of BARF1 to hCSF1 leads to an inactive conformation of hCSF1, hindering the interaction with its cognate receptor, CSF1R/CD115 (Figure 2). Therefore, BARF1 acts as an allosteric decoy receptor for hCSF1, interfering with macrophage activation and immune responses.

Using a recombinant BARF1 protein (BARF1.Fc) purified from the supernatant of monkey kidney cells transfected with plasmids containing the BARF1 gene fused to the Fc portion of human IgG1, Strockbine et al. demonstrated that the BARF1.Fc protein could bind to hCSF1, leading to suppression of the proliferative effects of hCSF1 on mouse bone marrow macrophages [21]. Later studies reported that recombinant BARF1 and BARF1 derived from EBV-infected B cells could inhibit IFN-α secretion from mononuclear cells [25]. Furthermore, hexameric sBARF1 protein could severely impair hCSF1-induced survival, proliferation and differentiation along with the oxidative and phagocytic function of macrophages [22]. Interestingly, an in vivo study using the rhesus macaque model with rhesus lymphocryptovirus (an EBV-related herpesvirus) revealed that BARF1 blockade of CSF1 signalling is an essential immune evasion strategy for both acute and chronic EBV infection [26]. 

In addition to the c-fms protein, sBARF1 was found to possess 18% sequence homology with the T-cell receptor co-stimulatory molecule CD80 [19]. Although there are structural differences between the N-terminal domains of CD80 and BARF1, the loops contacting the C-terminal domains and the loops facing the N-terminal domain are more conserved. Also, the binding surface of two N-terminal domains of the BARF1 dimer is similar to the dimerization interface between two CD80 monomers; however, there are no similarities for the interacting residues and the relative orientations of the Ig-like domains between CD80 and BARF1 [19]. No further studies have addressed the functional similarities between these two proteins. CD80 is a membrane glycoprotein present on antigen-presenting cells (APCs). Upon interaction with CD28 on T cells, CD80 promotes T cell differentiation into effector T cells. In contrast, CD80 also mediates an inhibitory interaction with CD152 (CTLA-4) to negatively regulate T cell activation [27]. EBV-associated NPC is characterized by a significant T lymphocyte infiltrate, although evidence suggests that the majority of T lymphocytes in the tumour microenvironment are functionally impaired, possibly as a consequence of IL-10 and TGFβ, which are induced by the EBV-encoded EBER1 and LMP1 proteins in NPC cells [28,29,30]. sBARF1 may also contribute to local immune modulation through interactions with CD28 and/or CD152, leading to a suppression in tumour infiltrating lymphocyte (TIL) function. This possibility, along with potential functional homology between BARF1 and CD80, is clearly an area worthy of further investigation.

### 2.2. The Mitogenic and Oncogenic Properties of BARF1

BARF1 has been shown to possess both mitogenic and oncogenic activity. Sall et al. demonstrated that the addition of purified sBARF1 to the culture medium of rodent fibroblasts, B cells and epithelial cells promoted cell cycle activation [31]. Similarly, the cellular uptake of sBARF1 was shown to induce G1/S phase cell cycle activation in human keratinocytes [32]. Also, sBARF1 purified from the serum of NPC patients or mice carrying BARF1-positive NPC xenografts was shown to promote B cell proliferation [33]. In addition, the ectopic expression of BARF1 has been demonstrated to induce morphological changes, to promote anchorage-independent growth and to induce tumourigenic transformation of mouse fibroblast lines NIH-3T3 and Balb/c-3T3 in newborn rodents [16]. Similar results were observed in HEK-293 human embryonic kidney epithelial cells, where BARF1 expression increased cell migration and anchorage-independent growth [9]. Although the underlying mechanisms utilised by BARF1 to induce cell immortalization and transformation are still largely unknown, BARF1 has been shown to immortalize primary monkey kidney epithelial cells through a mechanism involving c-Myc and activation of telomerase [34]. Mutational analysis has revealed that the first 54 amino acids of the N-terminus of BARF1 protein are essential for BARF1-mediated cell immortalization and malignant transformation. This region was also shown to upregulate expression of the cellular antiapoptotic Bcl-2 protein and to increase cell growth in soft agar and resistance to senescence upon serum deprivation [35]. Interestingly, the cleaved leader sequence peptide (aa 1–20) of BARF1 is within this region. The possible involvement of this cellular leader sequence in the oncogenic activity of BARF1 along with the putative cellular receptors for sBARF1 as well as the mechanisms and signalling pathways mediated by sBARF1 to induce gene expression, cellular immortalization and malignant transformation is worthy of further investigation.

Although BARF1 is expressed at extremely low levels in EBV-associated B cell lymphomas, BARF1 transcripts have been detected in EBV-positive cases of NKTL at levels equivalent to those observed in LCLs which can undergo some level of spontaneous lytic reactivation in vitro [12]. Ectopic expression of BARF1 in the EBV-negative B lymphoma cell line, Louckes, induces expression of c-Myc as well as several B cell “activation” antigens (CD71, CD21 and CD23) and, more importantly, an ability to form tumours in newborn rats and immune-compromised mice [15]. Interestingly, long-term culture of these cell lines resulted in a loss in BARF1 expression in certain clones, with a corresponding lack of tumourigenicity [15]. Similarly, BARF1 expression in an EBV-negative Akata BL cell line induced Bcl-2 expression as well as tumour formation in SCID mice [18]. 

As the expression of BARF1 is common in NPC and EBV-positive GC, it has been suggested that it plays an important, albeit undefined, role in EBV-driven epithelial oncogenesis [6,7,8]. A previous study has demonstrated that an NPC-derived EBV strain was capable of immortalizing primary monkey kidney epithelial cells in vitro. An analysis of viral gene expression in the cells revealed that this was limited to EBNA1 and BARF1, findings which suggest a role for BARF1 in cell growth transformation [36]. It was previously shown that infection of EBV-negative NPC cells with a recombinant EBV carrying the BARF1 gene under control of the SV40 promoter (BARF1-rEBV) resulted in higher cell growth density and greater resistance to apoptosis compared to cells infected with recombinant EBV (rEBV) which failed to express BARF1. Also, unlike their control rEBV-infected counterparts, BARF1-rEBV-infected NPC cells were tumourigenic in nude mice. These findings confirm the contribution of BARF1 to NPC tumourigenicity [37]. In EBV-positive GC cells, BARF1 has also been demonstrated to promote cell survival. Ectopic expression of BARF1 in an EBV-negative GC cell line stimulated the expression of genes associated with antiapoptosis and cell growth. BARF1 expressing GC cells also exhibited resistance to apoptosis induced by the anticancer drug, Taxol, and was accompanied by an increase in the expression ratio of Bcl-2 to Bax [38]. BARF1-mediated GC cell proliferation has been indicted to be mediated through reduction of p21 and SMAD4 as well as upregulation of NF-κB RelA, cyclin D1 and miR-146a [39,40]. 

Given that BARF1 is a secreted protein, the detection of intracellular BARF1 protein within EBV-positive cancer cells was proven to be difficult [6,39]. However, secreted BARF1 (sBARF1) is readily detected in the sera and saliva of NPC patients [33], indicating that sBARF1 is present in the bloodstream and body fluid of cancer patients. Interestingly, sBARF1 protein purified from the medium of 293T cells engineered to overexpress BARF1 could activate the cell cycle in rodent fibroblasts, human B cells and primary monkey kidney epithelial cells. These effects were specific as the sBARF1-induced growth-promoting effects could be blocked by anti-BARF1 antibodies [31]. Similarly, siRNA-mediated silencing of BARF1 in EBV-positive BL and NPC cells resulted in inhibition of cell growth and induction of caspase-dependent apoptosis [41]. These findings suggest that targeting BARF1 may constitute a viable therapeutic strategy for EBV-driven malignancies, particularly EBV-associated carcinomas.

### 2.3. The Therapeutic Potential of BARF1

#### 2.3.1. BARF1 Sequence Conservation 

Sequence variation in the BARF1 gene has been identified in Indonesian NPC tumours. The primary mutations that cause amino acid substitutions at position V29A, W72G and H130R were observed [42]. All three mutations do not affect the tertiary structure of the hexameric sBARF1 complex. The V29A mutation is also dominant in northern Chinese NPC tumours and has a higher frequency in NPC cases (25%, 20/79) than in EBV-positive GC cases (0%, 0/45) and healthy controls (4%, 2/46) [43]. Although V29A is located within the first 54 residues of the amino-terminal transforming domain of BARF1, functional studies indicate that the V29A mutation does not influence cellular transformation [43]. Using a larger sample size (*n* = 293), Liu et al. found that only 16% (23/141) of NPC tumours obtained from southern and northern China harboured the V29A mutation [44]. Instead, a silent mutation at amino acid 14 was identified as the most frequent mutation in NPC tumours (44%, 62/141) compared to healthy controls (16%, 24/152). Additional mutations have also been found in NPC tumours; however, none of these are located within the functional domains of BARF1. These findings suggest that the sequence of BARF1 is highly conserved, making this viral protein a good candidate for targeted therapies. 

#### 2.3.2. T Cell-Based Immunotherapy

Clinical evidence indicates that adoptive cell transfer immunotherapy (ACT) is an effective and safe approach for treating EBV-associated malignancies. ACT is based on the use of autologous EBV-transformed LCLs as antigen-presenting cells (APCs) to induce EBV-specific cytotoxic T lymphocytes (CTLs) in vitro. The clinical utility of ACT has been substantiated in posttransplant lymphoproliferative disorder (PTLD), where the full repertoire of EBV latency-associated proteins are expressed [5,45]. However, the outcomes for patients with EBV latency II associated-malignancies are not promising, with only a fraction of HL and NPC patients receiving ACT showing clinical responses [46,47,48,49]. This lack of clinical responsiveness may be due to the restricted number of viral proteins expressed in tumour cells or to the fact that only a minor fraction of the CTLs generated from LCLs can recognize these viral proteins. Also, the EBV proteins (LMP1, LMP2 and EBNA1) expressed in NPC cells are known to be poorly immunogenic [50,51]. To overcome this limitation, APCs have been genetically engineered to present specific EBV type II antigens or specific peptide epitopes to generate CTLs. In phase I/II clinical studies for NPC, NKTL and HL patients, adoptive transfer of CTLs specifically recognizing LMP1, LMP2 or EBNA1 has shown promising antitumour activity [46,52,53,54]. 

To broaden the specificity and to boost the antitumour activities of EBV-specific CTLs, additional EBV proteins must be included in the repertoire of antigens required for CTL generation. In addition to LMP1, LMP2 and EBNA1, BARF1 is an attractive candidate given that cellular immune responses to BARF1 have been described in patients with EBV-associated malignancies. Using IFNγ-ELISpot (enzyme-linked immunospot) assay, spontaneous CD4+ and CD8+ T cells specific for sBARF1 protein were identified in the peripheral blood from both EBV-seropositive donors and NPC patients but not in EBV-seronegative controls [55]. Furthermore, CD8+ T cells specific for peptides derived from the N-terminal region of BARF1 could be expanded from peripheral blood mononuclear cells (PBMCs) of both EBV-seropositive healthy individuals and NPC patients. Interestingly, T cell responses to BARF1 were always higher in NPC patients than in healthy individuals. Moreover, autologous monocyte-derived dendritic cells pulsed with BARF1-derived peptides could induce BARF1-specific CTLs and these could recognize and lyse cancer cells endogenously expressing the BARF1 protein [55]. Similar findings were observed in a recent study in which an unbiased, peptide library approach was used to characterize BARF1-specific T responses in EBV-seropositive healthy donors and patients of NPC and NKTL [56]. BARF1-specific CD4+ and CD8+ T cells were identified in approximately 70% of healthy donors and cancer patients. BARF1-specfic T cells recognized both Major histocompatibility complex (MHC) class I and II epitopes clustered in the N-terminal region of BARF1. Also, T cells specific for BARF1 epitopes could be reactivated and expanded from peripheral blood of EBV-seropositive individuals and could recognize and kill EBV-positive cancer cells [56]. Findings from these two studies indicate that BARF1 is naturally immunogenic for T cells and that BARF1-expressing cancer cells can process and present BARF1 peptides (located in N-terminal region) as natural epitopes for CTL recognition and killing. Also, BARF1-specific CTLs can be generated in vitro from the blood of EBV-seropositive individuals. In this regard, targeting BARF1 has the potential to improve the efficacy of current T-cell immunotherapy approaches for EBV-driven malignancies.

To enrich the number of BARF1-specific CTLs generated for ACT, Fae et al. developed a protocol to induce the expression of lytic BARF1 protein in LCLs [57]. This protocol is based on the use of low doses of doxorubicin to trigger lytic replication in LCLs, allowing cells to effectively express BARF1 protein and to present BARF1 peptides for CTL reactivation. This protocol has been proven to efficiently generate BARF1-specific effector T cells from both healthy donors and cancer patients. Given that doxorubicin is a conventional chemotherapy drug for which safety and effectiveness have been tested, the concept of using doxorubicin to increase BARF1 peptide presentation in LCLs/APCs further increases the applicable potential of BARF1 for immunotherapy of EBV-driven malignancies.

#### 2.3.3. Antibody-Based Immunotherapy

In addition to BARF1-specific T lymphocytes, antibodies against BARF1 have been detected in the sera of both acute and chronic infectious mononucleosis (IM) patients and NPC patients [17]. Using an immunofluorescence-based staining assay incorporating recombinant BARF1 protein, 84.8% NPC patients (*n* = 125) were shown to have detectable, albeit low titres of IgG specific for the BARF1 protein [58]. Similarly, using purified native sBARF1 protein as an antigen in antibody capture ELISA analysis, IgG responses to BARF1 were found in the serum of both NPC patients and healthy EBV carriers, although NPC patients had stronger responses compared to healthy seropositive individuals. However, IgG reactivity to BARF1 was found to be weak in comparison to other EBV proteins, VCA and EBNA1 [59]. 

Although poorly immunogenic, the presence of antibodies against BARF1 in EBV-positive cancer patients suggests that it constitutes a viable target for antibody-based immunotherapy. In a recent study, a novel BARF1-specific monoclonal antibody (3D4) was developed for immunotherapeutic purposes [60]. 3D4 recognises an epitope (aa28–35) located at the N-terminal domain of BARF1 and close to the binding site at residues 34–39 for hCSF1 [24]. This monoclonal antibody (mAb) can influence the interaction of BARF1 with hCSF1, an effect that can potentially relieve the inhibitory effects of BARF1 on macrophage differentiation. In vitro studies had shown that the 3D4 mAb recognised the BARF1 protein in both EBV-positive lymphoma and GC cells. The 3D4 mAb also prove useful in complement-dependent cytotoxicity (CDC) and antibody-dependent cell-mediated cytotoxicity (ADCC) assays in a panel of BARF1-positive tumour cells derived from NHL, BL, NPC and GC. Furthermore, biodistribution analysis of a fluorescently labelled 3D4 mAb in mice bearing EBV-positive NPC xenografts revealed that the mAb could identify BARF1 expressing tumour masses in vivo. Also, 3D4 mAb treatment actively restrained tumour growth and the dissemination of BARF1-positive NPC and lymphoma xenografts, an effect that could improve the survival of mice bearing tumours [60]. Unlike EBV-specific CTL therapy that only targets antigens presented at the cell surface of APCs, BARF1-specific mAbs recognise and block sBARF1 protein, thus potentially hampering the mitogenic and immunomodulating activity of sBARF1 in the tumour microenvironment. The clinical use of 3D4 mAb alone or in combination with radiotherapy and chemotherapy for the treatment of EBV-driven malignancies, particularly NPC and GC, is an area worthy of further investigation.

#### 2.3.4. Implication of Using Small Molecule or Peptide to Target BARF1 for Therapeutic Intervention

In addition to EBV-specific antibody and CTL-based immunotherapies, other therapeutic strategies that use small molecules or peptide-based inhibitors to reactivate virus replication or to inhibit the oncogenic function of viral proteins are being developed. For example, peptides derived from EBNA3c and EBNA2 have been shown to inhibit EBV-mediated primary B cell immortalization and hyperproliferation and to suppress lymphoblastoid outgrowth from PBMCs in culture [61,62]. In addition, inhibiting EBNA1 with dominant-negative EBNA1 mutants, EBNA1 antisense oligonucleotides and EBNA1 siRNA have been found to cause termination of latent EBV infection and an ablation of the effects of EBV in malignant and non-malignant diseases [63,64,65,66]. 

sBARF1 mediates its immunomodulatory activities by binding to the dimer interface of hCSF1 and possibly through interactions with CD28 and CD152 [19,23,24]. Therefore, BARF1 may constitute a viable candidate for peptide-based therapy. Recently, our group has engineered two EBNA1-specific probes, L2P4 and UCNP-P4 [67,68]. The L2P4 probe contains a water-soluble fluorophore L2 and an EBNA1-binding peptide, P4 (YFMVF-GG-RrRK), incorporated with a nuclear localization sequence (RrRK) moiety, which allows nucleus penetration and localization. P4 can occupy the first EBNA1 dimerization interface within the DNA-binding domain and can interfere EBNA1 homodimerization, preventing EBNA1-mediated replication and transcription. UCNP-P4 is a signal-enhancing and peptide-stabilized probe which contains upconversion nanoparticles (UCNP) and the EBNA1-binding peptide, P4. UCNP-P4 aggregates upon binding to EBNA1, increasing a responsive upconversion signal for imaging. Both these probes have been shown to inhibit the growth of EBV-positive NPC cells in culture and NPC tumour xenografts in nude mice. This approach of peptide design for EBNA1 interference may also be applied to the BARF1 protein. With a BARF1-specific peptide designed to block the binding site of BARF1 to hCSF1 and possibly other molecules, the function of BARF1 in immunomodulation may be inhibited. Given that sBARF1 is a secreted protein, a fluorescent peptide probe specific for BARF1 would prove useful to track the intra- and extracellular distributions of sBARF1 and to aid our understanding of the mechanisms involved in sBARF1-mediated oncogenicity and immunomodulation.

Our group has also constructed an EBNA1 and Zn^2+^ chelating probe (ZRL5P4) which can selectively disrupt EBNA1 homodimerization, structure and functions; can emit unique responsive fluorescent signals; can label EBV-positive cells; and can inhibit the in vitro and in vivo growth of EBV-infected cells. Most importantly, the treatment of NPC cells with ZRL5P4 can disrupt EBV latency and reactivate EBV lytic cycle through the induction of early and late EBV lytic proteins [69]. Given that BARF1 is a lytic protein in EBV-infected B cells, it will be interesting to determine not only whether combinations of the ZRL5P4 and BARF1-specific peptide probes induce EBV lytic reactivation through the disruption of EBNA1 and BARF1 activities but also whether this combination proves to be an effective therapeutic approach for the treatment of EBV-driven lymphomas.

## 3. Concluding Remarks

The BARF1 protein possesses various immunomodulatory and oncogenic properties. It interferes with the function of hCSF1, thereby inhibiting macrophage differentiation, activation and IFN-α secretion. BARF1 not only blocks cell apoptosis but also can promote cell growth, immortalization and malignant transformation through upregulation of the NF-κB pathway and the induction of genes associated with cell cycle control and cell death [9,18,34,35,39,40]. As a secreted protein, BARF1 is found in the body fluids of patients with EBV-associated cancers. Although weakly immunogenic, elevated humoral and cellular immune responses to BARF1 have been documented in patients with EBV-associated malignancies, suggesting that BARF1 constitutes a good candidate for both antibody-based and T cell-based immunotherapy. From an immunotherapeutic perspective, the BARF1-specific monoclonal antibody, 3D4, has shown promise in neutralising BARF1 function in vitro and in vivo, while a protocol of inducing lytic reactivation in LCLs to enhance the production of BARF1-specific CTLs has been developed [57,60].

Given that the N-terminal region of BARF1 is responsible for binding hCSF1 and BARF1-mediated cell growth enhancement and malignant transformation, future studies are required to determine whether agents which target the BARF1 N-terminus specifically are able to block the oncogenic and immunomodulatory functions of the BARF1 protein [23,24,35]. Our group has developed EBNA1-specific fluorescent peptide probes (L2P4 and UCNP-P4) which target the EBNA1 dimerization site [67,68]. These probes have proved successful not only in the imaging of tumour xenografts in vivo but also in suppressing the growth of EBV-positive tumour cells in vitro and in vivo. A similar approach may also be applied to the BARF1 protein. BARF1-specific peptides conjugated with an appropriate fluorophore may be useful in both the tracking of BARF1-expressing tumours in vivo and, more importantly, in neutralising BARF1 function. In time, such reagents may help us to understand the mechanism(s) by which BARF1 mediates its oncogenic and immunomodulatory activities and its role in EBV-associated malignancies. It will be interesting to investigate whether combinations of BARF1-specific peptide probes in conjunction with the newly constructed EBNA1 and Zn2+ responsive probe (ZRL5P4) both to stimulate lytic reactivation and to inhibit EBNA1 and BARF1 might prove useful for the treatment of EBV-associated lymphomas [69].
